# Replacement of a Tooth

**Published:** 1868-07

**Authors:** 


*Replacement of a Tooth.*—On the 16th of June last we were called on to extract a superior second bicuspid tooth for a gentleman, who is at the present time a student in our office, the history of which will no doubt prove interesting to our readers.

This tooth was, by mistake, extracted in 1845 on the morning of one day, and on the evening of the succeeding day returned to its cavity, where it has remained and proved a useful organ for a period of nearly twenty years. At the time we removed it, the gum had receded on the posterior approximal surface of the root, for some distance from the neck of the tooth, but the attachment was so strong on the remaining portion of the root, as to require considerable force to remove it from its cavity. It had at times within the past twelve months become a source of irritation, but previous to this had given no trouble whatever.

